# Floral Reward, Advertisement and Attractiveness to Honey Bees in Dioecious *Salix caprea*

**DOI:** 10.1371/journal.pone.0093421

**Published:** 2014-03-27

**Authors:** Stefan Dötterl, Ulrike Glück, Andreas Jürgens, Joseph Woodring, Gregor Aas

**Affiliations:** 1 Department of Plant Systematics, University of Bayreuth, Bayreuth, Germany; 2 Ecological-Botanical Garden, University of Bayreuth, Bayreuth, Germany; 3 School of Biological and Conservation Sciences, University of KwaZulu-Natal, Pietermaritzburg, South Africa; 4 Department of Animal Ecology, University of Bayreuth, Bayreuth, Germany; Monash University, Australia

## Abstract

In dioecious, zoophilous plants potential pollinators have to be attracted to both sexes and switch between individuals of both sexes for pollination to occur. It often has been suggested that males and females require different numbers of visits for maximum reproductive success because male fertility is more likely limited by access to mates, whereas female fertility is rather limited by resource availability. According to sexual selection theory, males therefore should invest more in pollinator attraction (advertisement, reward) than females. However, our knowledge on the sex specific investment in floral rewards and advertisement, and its effects on pollinator behaviour is limited. Here, we use an approach that includes chemical, spectrophotometric, and behavioural studies i) to elucidate differences in floral nectar reward and advertisement (visual, olfactory cues) in dioecious sallow, *Salix caprea*, ii) to determine the relative importance of visual and olfactory floral cues in attracting honey bee pollinators, and iii) to test for differential attractiveness of female and male inflorescence cues to honey bees. Nectar amount and sugar concentration are comparable, but sugar composition varies between the sexes. Olfactory sallow cues are more attractive to honey bees than visual cues; however, a combination of both cues elicits the strongest behavioural responses in bees. Male flowers are due to the yellow pollen more colourful and emit a higher amount of scent than females. Honey bees prefer the visual but not the olfactory display of males over those of females. In all, the data of our multifaceted study are consistent with the sexual selection theory and provide novel insights on how the model organism honey bee uses visual and olfactory floral cues for locating host plants.

## Introduction

Only about 6% of flowering plants worldwide have separate male and female individuals, a phenomenon called dioecy [1], [2]. Dioecious plant species are rare when compared to species with hermaphrodite flowers, possibly because the risk of reproductive failure is relatively high due to their complete dependence on the presence of pollinating agents. Moreover, dioecious (as is true for monoecious species) and animal-pollinated plants must ensure that potential pollinators are attracted to both sexes and switch between individuals of both sexes regularly in order to transfer pollen. Spatial separation of sexes does result in differing flower morphology because of the presence or absence of functional gynoecia and androecia. Differences between males and females may result in attracting different flower visitor species or having different visitation rates [3]–[5]. While the former is clearly disadvantageous as it prevents pollen transfer from males to females, the latter may have neutral, positive, or negative effects on the male and female function, respectively. As males can offer both pollen and nectar, and females only nectar, visitation rates to female flowers often are lower than to male flowers [6]–[9]. It was hypothesised that this is not a disadvantage because the female function (pollen receipt) generally requires lower visitation rates than the male function (pollen dispersal) [10]–[13]. Historically, male fertility has been suggested to be limited by access to mates, whereas female fertility is (in plants due to the production of fruits) rather limited by resource availability (Bateman's principle, [14]). According to the sexual selection hypothesis, male flowers are therefore suggested to invest more in traits associated with pollinator attraction than female flowers [15]–[17]. However, the assumption that only male fertility is limited by access to mates is not generally true because female fertility also can be limited by access to mates (through pollen limitation) [18], [19], and low visitation rates of female flowers may also result in a lower reproductive success [1], [20]–[22]. Therefore, in some plants, female pollen-lacking flowers are thought to mimic pollen-offering male flowers to enhance visitation and reproductive success, and avoid flower visitors' specialization for either male or female flowers [20], [23]–[25]. Depending on which sex is more limited by access to mates, either males or females should be under higher selective pressure to increase their attractiveness; only if both sexes are similarly limited by access to mates, are they predicted to be similarly attractive [15]. Consequently, in addition to basic morphological differences, sex-specific selection has often led to further divergence between sexes with respect to physiology [26], flower size [9], and other aspects of floral advertisement and reward [5], [27]–[30], in order to enhance male and female reproductive efficiency [31]. In many dioecious species, nectar is the only reward both sexes have in common, and if nectar composition or quantity differs between the sexes, it may influence pollinators' preference for one sex or the other, especially through learning.

Advertising through visual and/or olfactory cues is important to attract pollinators to the floral rewards of either sex [32], [33]. However, few studies are available comparing visual or olfactory cues quantitatively between the sexes [15], [34], [35], and little is known about how flower visitors respond to differences in the visual and olfactory floral advertisement. Ashman et al. [36] and Ashman et al. [37] demonstrated in gynodioecious wild strawberry (*Fragaria virginiana* Mill.), that higher visitation rates of hermaphroditic compared to female flowers are due to a number of cue differences, although a special scent emitted from anthers was most responsible for the preference of pollinators for hermaphroditic flowers. In studies on dioecious *Silene latifolia* Poir., male flowers were found to emit higher amounts of scent than females [17]. Male *Silene* flowers were innately more attractive for males but not females of its nursery pollinator, the moth *Hadena bicruris* (Hufn.) [17]. Similarly, Theis et al. [38] found higher amounts of scent in male compared to female inflorescences of *Cirsium arvense* (L.) Scop., and male flower heads were preferred by some bees. In both the *Silene* and the *Cirsium* studies, the pollinators responded to natural flowers, and it is unclear, whether the choices of pollinators were guided by olfactory cues only, or also by visual ones.

As part of a long term study on the pollination biology of dioecious willow species (*Salix* L., Salicaceae), we are interested in both differences in floral reward and advertisement (visual, olfactory cues) of male and female willow inflorescences and their effect on attraction, guidance and behaviour of its pollinators [39]–[41]. *Salix* L. is a genus of woody dioecious plants with numerous species [42], [43] with a nearly global distribution. Willows are mostly entomophilous, although in some species wind also contributes to a variable extent to pollination [44]–[46]. The flowers are arranged in catkins, whereby male catkins offer pollen and nectar as a reward, and female inflorescences offer nectar only. For our investigations we chose *Salix caprea* L. (sallow), which is one of the most common *Salix* species in Central Europe. Although flowering very early in the year, at the end of winter towards the onset of spring (March/April), insect pollination is predominant, even though wind pollination may account for up to 50% of seed set [46], [47]. *Salix caprea* is visited by many insect species, including social and solitary bees, flies, butterflies, moths and beetles, however, one of the most frequent visitors is the honey bee (*Apis mellifera* L.) [46], [48], [49]. Although the percentage number of honey bees switching from male to female catkins is low [49], they act as important natural pollinator and a single visit to a catkin results in a seed set of on average 4%, reaching maxima of up to 11% (Nathalie Moske, personal communication).

Here, we analysed the visual and olfactory advertisement of female and male flowers/flowering twigs, determined their relative importance in attracting honey bees, and studied properties of nectar, the common reward offered by both sexes. We ask, 1) what is the relative importance of the visual and olfactory advertisement in attracting inexperienced honey bees to sallow flowering twigs?, 2) do female and male flowers/flowering twigs have the same or a different visual and olfactory advertisement?, 3) do visual and olfactory cues of female and male flowering twigs have the same attractiveness for inexperienced honey bees or is one sex initially more attractive than the other?, and 4) does nectar have the same or different properties in female and male flowers? Our results show that the amount of nectar reward is the same, but sugar composition differs between the sexes. A combination of visual and olfactory sallow cues was most attractive to honey bees, followed by olfactory cues and finally visual cues. Male flowers are more colourful than females, emit a higher amount of scent than females and are preferred by honey bees over those of females due to differences in the visual advertisement.

## Materials and Methods

### Ethics Statement

The Botanical Garden is on the campus of the University of Bayreuth and we were allowed to perform the sampling.

### Plant Material

All *Salix caprea* plants used in this study were located in the Ecological-Botanical Garden (EBG) in Bayreuth, Germany.

### Collection and Analysis of Nectar

In 2006, 25 pooled nectar samples were collected from open-pollinated, unbagged flowers of fully blooming catkins of 11 female and 14 male individuals of *Salix caprea*. Sampling took place between 1100 h and 1400 h on sunny days with at least 10°C air temperature. Nectar samples were collected with 0.5 µL capillaries (“Minicaps”, Hirschmann Laborgeräte, Eberstadt, Germany), each sample from five to 15 young flowers of a single catkin. Pooling of nectar was necessary because the amount of nectar per flower was too low for separate collection. Furthermore, in *S. caprea*, the number of catkins per plant individual is highly variable in both sexes, and depends on the size and age of the plant (unpublished data). The number of flowers within a catkin is also highly variable and we therefore did not calculate the amount of nectar or sugars per catkin or per plant. Nectar volume per sample and per flower (volume in the pooled samples was divided by the number of flowers used) was determined and nectar was transferred into Eppendorf reaction tubes with 200 µL Milli-Q-Water and immediately frozen at −80°C until further analysis.

The samples were analysed using high performance liquid chromatography. The HPLC Jas.co PU-1580 (JASCO GmbH, Groß-Umstadt, Germany) was equipped with a CarboPac PA 100, 4×250 mm column (Dionex Corp., Sunnyvale, California), and an electrochemical detector (Dionex ED 40; Dionex Corp., Sunnyvale, California). Frozen nectar samples were thawed and diluted from 1∶10 to 1∶100 with Milli-Q-Water, and a 2 µL subsample was injected for analysis. Samples were eluted with a gradient from 3 to 70% 0.5 M NaOH at a flow rate of 1 mL min^−1^. The process was controlled with Borwin Chromatogram software, which generated the chromatograms. The sugar content was determined by comparison with standards (glucose, fructose, and sucrose), and for each sample, the average amount of sugar per single flower (µg), and the relative sugar composition (percentage amount of the single sugars) were calculated.

In 2011, we collected another 21 nectar samples as described above (this time using 1 µl capillaries “Minicaps” from Hirschmann Laborgeräte, Eberstadt, Germany) from 6 males (subset of individuals used 2006) and 15 females (11 individuals from 2006+4 additional individuals) and determined the sugar concentration as sucrose equivalents (%, w/w) using a refractometer (Bausch and Lomb, ABBE-3L).

Using STATISTICA [50], Mann-Whitney-U tests were calculated to test for differences in nectar properties between the samples collected from male and female individuals.

### Colorimetric Measurements and Analyses

We used quantitative colorimetry to characterise the colours of male and female flower parts, and colour modelling to determine how the colours are perceived by the honey bees. The spectral reflectance properties of pollen, filaments, stigmatic lobes, ovaries (willow flowers are strongly reduced and do not have a perianth), and emerging green leaves available during flowering (for comparison), were determined with a double beam Varian Cary 5 (Varian, Inc, Palo Alto, California) spectrophotometer. We used a barium sulphate white standard and the Praying Mantis Accessory (Harrick Scientific Products, Inc., Pleasantville, New York). The sample disk was wrapped in black electrical tape with the sticky side out, and flower parts and pollen (collected from open anthers) were pressed onto the tape [32]. Data obtained from these measurements were subsequently used to plot the colour loci of the different flower parts in the colour hexagon against the colour loci of the leaves (which did not differ between the sexes). Honey bees are UV-Blue-Green trichromats [51], [52] and this colour diagram visualizes how they perceive the flowers, and allows calculating colour distances among the different samples [32], [53]. Behavioural experiments with bees trained to visit artificial flowers demonstrated that colour distances smaller than 0.05 hexagon units are poorly discriminated, whereas distances of 0.10 or more are well discriminated [51].

### Collection and Analysis of Floral Scent

In the flowering seasons of 2006 and 2007 scent samples were collected using two different dynamic headspace methods. In 2006 the floral scent of six male and five female individuals was collected *in situ* following Füssel et al. [41]. Scent was sampled at 1400 h from one twig containing 4 to 10 flowering catkins from each individual. In 2007, scent was sampled from cut twigs of male (four samples) and female (four samples) individuals, immediately after they had been used as olfactory cues in the behavioural experiments (*Experiment 3*, see below). Those twigs originated from a different set of individual plants than was used in 2006. To distinguish between plant volatiles and ambient contaminants, the surrounding air was collected for comparison in 2006 as well as 2007.

The catkins were enclosed for 10 min in an oven bag (Nalophan), and the floral scent was subsequently trapped in an adsorbent micro tube (filled with 1.5 mg of Tenax-TA 60–80 and 1.5 mg of Carbotrap 20–40; Supelco, Bellefonte, Pennsylvania) using a membrane pump (G12/01 EB, Rietschle Thomas, Puchheim, Germany) for 2.5 min. This bagging method and duration of scent collection were found to give strong samples without saturating the adsorbent tubes or the mass spectrometer. Scent molecules were identified using a combination of gas chromatography and mass spectrometry (GC-MS) as described earlier [41]. A Varian (Varian, Inc., Palo Alto, California) Saturn 2000 mass spectrometer and a Varian 3800 gas chromatograph (column: ZB-5, 60 m length, 0.25 mm inner diameter, 0.25 µm film thickness; Phenomenex, Inc., Torrance, California) with a 1079 injector that had been fitted with the ChromatoProbe kit [54] was employed. The micro tube was loaded into the probe, which was then inserted into the modified GC injector. The injector split vent was opened and the injector heated to 40°C to flush any air from the system. The split vent was closed after 2 min and the injector was heated to 200°C, and held at this temperature for 4.2 min, after which the split vent was opened and the injector cooled down. The GC oven temperature was held for 7 min at 40°C, then increased by 6°C per min to 250°C and held for 1 min. The MS interface was 260°C and the ion trap was set to 175°C. The mass spectra were taken at 70 eV (in EI mode) with a scanning speed of 1 scan/s from *m/z* 30 to 350. The compounds were identified as described in Füssel et al. [41]. For each compound, the percentage of the total peak area in each sample was calculated. We estimated total scent emission (absolute amount) by injecting known amounts of lilac aldehydes, *trans*-β-ocimene, *cis*-3-hexenylacetate, benzaldehyde, phenylacetaldehyde, and veratrole and used the mean response of these compounds for quantification [55].

The Bray-Curtis index was calculated (PRIMER 6.1.11 package) to determine semiquantitative differences in floral scent patterns between male and female individuals. For these analyses, the percentage amounts of the floral scent compounds were used. To visualise the similarities/dissimilarities in floral scent patterns among samples a nonmetric multidimensional scaling was used [56].

To test for differences in scent between the sexes, we calculated in PRIMER, on the basis of the Bray-Curtis similarity matrix, a PERMANOVA analysis [57] with the fixed factors sex and year in a crossed design. The factor year was included to test whether the sex effects were influenced by the different methods used in the two different years (cut twigs versus *in situ*). PERMANOVA is a technique for testing the simultaneous response of one or more variables to one or more factors in an ANOVA experimental design on the basis of a (dis)similarity matrix, using permutation methods [57].

Similarity percentage (SIMPER) was used, again in the PRIMER package (two way crossed design; factors: sex, year) to determine the compounds responsible for differences between sexes.

### Bioassays

**Figure 1:**
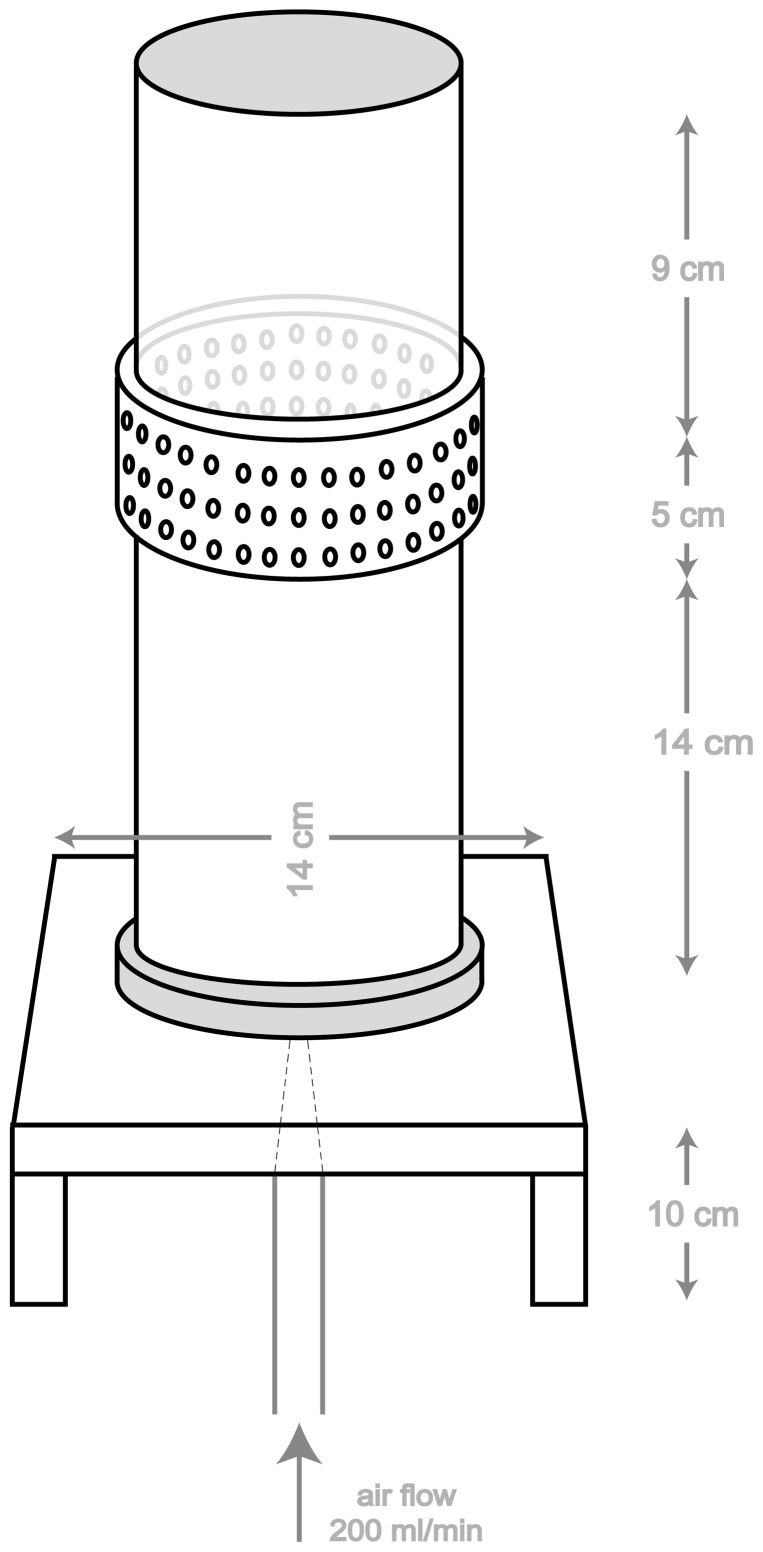
Basic construction of quartz glass cylinders used in the behavioural experiments. Transparent cylinders with a macrolon sleeve that contained holes was used to offer visual + olfactory cues, a cylinder without the holes in the macrolon sleeve was used to offer only visual cues, and a black cylinder with a macrolon sleeve that contained holes was used to offer only olfactory cues.

To compare the attractiveness of a combination of visual and olfactory cues, of decoupled visual and olfactory cues, and of male and female *S. caprea* to *A. mellifera*, several dual-choice bioassays were performed. In the bioassays, bees were offered flowering sallow twigs in quartz glass cylinders (did not block light in the visible range of the bees) constructed to provide either visual or olfactory cues only, or both. The cylinders were the same as described before [58]–[60] and the basic construction of such a cylinder is shown in Figure 1. As negative controls we used empty cylinders identical to the cylinders containing the sallow cues to be tested.

**Figure 2:**
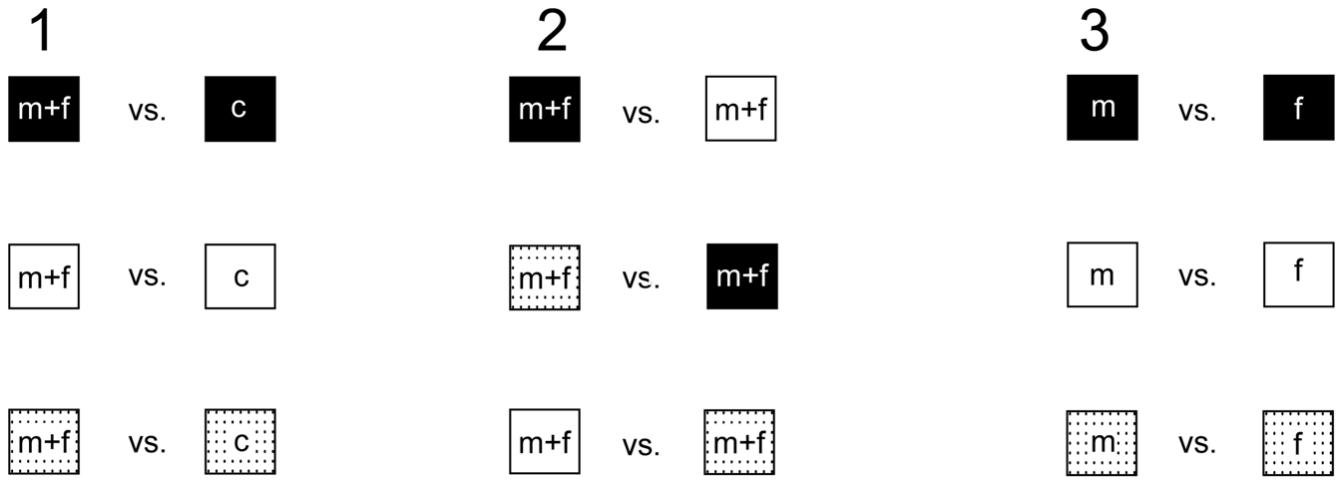
Cylinder arrangement of the three experiments performed. (1) Attractiveness of different floral traits against a negative control, (2) relative attractiveness of the different floral traits against each other, (3) attractiveness of males against females. Filled squares = olfactory traits; open squares = visual traits; dotted squares = olfactory and visual traits combined; squares with c (control) = empty cylinders; m = male twigs; f = female twigs.

Three different experiments were conducted (Figure 2). In *Experiment 1* we used a combination of male and female flowering twigs and compared them to negative controls (no twigs) with regards to the attractiveness of visual cues alone, olfactory cues alone, and a combination of both cues. In *Experiment 2* we again pooled male and female flowering twigs and tested visual against olfactory cues, and a combination of both cues against either visual or olfactory cues. In *Experiment 3* we tested male and female flowering twigs separately and compared the sex specific attractiveness of visual and olfactory cues alone or in combination.

Bioassays were performed during the flowering season from March 12^th^ to March 30^th^ 2007. Flowering twigs were cut in the field and the cut ends were wrapped in moist tissue paper and placed in oven bags to prevent scent emission from damp tissues. In all tests of *Experiments 1* and *2*, four female (from one individual) and four male (from one individual) flowering twigs including the emerging green leaves were enclosed together in one cylinder. Each cylinder had altogether approximately 80 catkins. In all tests of *Experiment 3*, either eight male or eight female twigs with approximately 80 catkins were enclosed in a cylinder. In nature, willows often grow in dense patches, females and males intermingled, and bees approaching such a patch need to decide which sex to visit.

The dual-choice bioassay was performed in a flight cage (7.20 m×3.60 m×2.20 m) set up in a greenhouse [61]. During the experiments the side windows (2×15 m^2^) and the roof (2×20 m^2^) were opened, allowing natural light to enter without being filtered through the glass. A hive with nine frames of *A. mellifera* was placed in the flight cage two weeks before the first flowers of *Salix* opened. Until the beginning of the experiment on March 12^th^, the bees were fed a sugar solution. These bees are designated as naïve. The sugar solution feeder was positioned at the region where later the test cylinders for the bioassay were set up, in a way that the distance to both cylinders was the same. For each experiment both test cylinders were set up 3 m away from the bee hive and 1 m away from each other. All experiments were performed only on sunny days with at least 10°C air temperature between 1200 h and 1500 h when the activity of bees was high. Each test was conducted for a total of 40 min, whereby after 20 min the position of the two cylinders was exchanged. Each test was repeated once, with material from other plants. The responses of the bees were classified in two categories: (1) “zigzagging” (*Z*), if bees flew “zigzag” to within 10 cm of a cylinder, hovered in front of it without landing, and (2) landing (*ZL*) after zigzagging and hovering. Thus, *Z+ZL* is the total number of bees zigzagging whether they landed or not. It was not possible to catch all the responding bees but we tried to avoid counting a bee more than once in a particular dual-choice test. One individual bee may have participated in different dual-choice tests. However, as bees were not rewarded at the cylinders they still were naïve according to Lunau [62] though the absence of a reward may have taught them to avoid the stimulus.

To compare the numbers of bees that showed a specific response (Z, ZL, Z+ZL) to the different cylinders in a particular dual-choice test, an observed vs. expected Chi-Square (χ^2^) test [50] was conducted on the pooled data of the two replicates, but only, if the expected frequencies were greater than five.

## Results

### Floral Nectar

Female and male flowers offered the same nectar volumes and total sugar amounts (Table 1). Nectar of both male and female flowers contained three sugars: fructose, glucose, and sucrose, but sugar composition differed significantly. Nectar from males was dominated by sucrose (89%; min-max: 67%–98%) and contained only small amounts of fructose and glucose, while nectar from females contained similar amounts of the three sugars. The sugar composition was hexose-rich in females but sucrose-rich in males.

**Table 1:** Nectar characteristics of male and female *S. caprea*.

	Male catkins			Female catkins			MW-U-Test
	Median	Minimum	Maximum	Median	Minimum	Maximum	Significance
Nectar volume per flower (µL)	0.010	0.005	0.017	0.012	0.005	0.027	ns
Sugar concentration (%, w/w)	38.3	25.9	59.0	48.6	26.6	73.4	ns
Glucose (%)	5.5	0.1	19.4	35.1	24.8	56.8	***
Fructose (%)	5.6	1.8	15.7	32.0	4.6	44.5	***
Sucrose (%)	88.9	66.7	98.1	32.9	7.0	52.1	***

Nectar volume and relative amount of single sugars were determined in 14 male and 11 female *S. caprea* plants (2006 samples). The sugar concentration was determined in samples from other 6 males and 15 females (2011 samples). The significance of sex differences between nectar samples are given according to Mann-Whitney-U-tests (***: *p*<0.001; ns: p>0.10).

### Flower Colour

**Figure 3:**
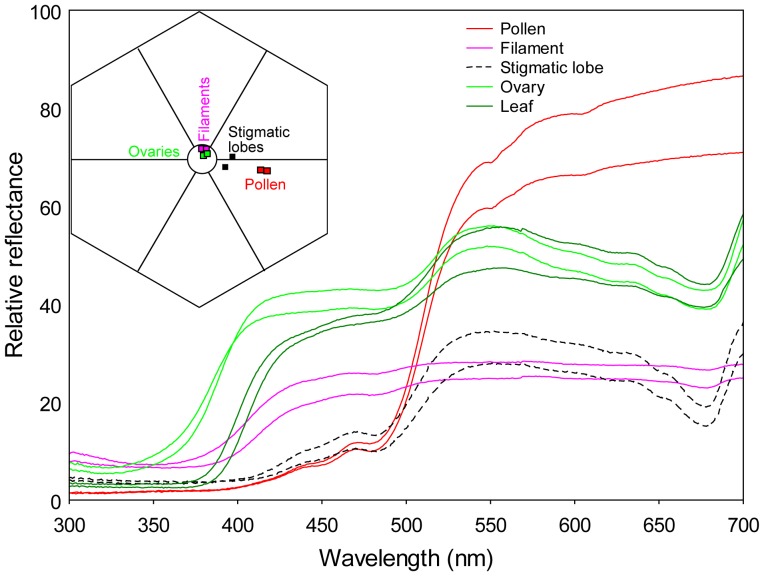
Spectrophotometric measurements and bee colour space. Spectral reflectance functions of the two replicate measurements of leaves and different flower parts (F: filament; O: ovary; P: pollen; S: stigmatic lobe) of *Salix caprea*, and corresponding colour loci plotted in a hexagon colour space against the leaves. E: Excitation. Honey bees cannot discriminate the filaments and the ovaries, but the stigmatic lobes and pollen, from the leaves. The distance in the hexagon between the pollen and the stigmatic lobe samples ranges from 0.21 to 0.26 units.

The colorimetric measurements (Figure 3) revealed that the ovaries in female flowers and the filaments in male flowers have a similar reflectance as the leaves of *S. caprea*, which are densely covered by whitish hairs during the time of flowering and therefore are greenish-whitish. In contrast, both stigmatic lobes and pollen can be discriminated against the background of leaves. However, the colour of pollen clearly differs from the colour of stigmatic lobes. The pollen, which is presented on the surface of the open anthers topping the filaments, is bright yellow (bee green) and reflects light mainly from 500 nm to 700 nm, whereas the stigmatic lobes are yellow-greenish. In the colour hexagon the distances between the pollen and the style samples range from 0.21 to 0.26 units. The distances between the pollen loci and the uncoloured point (centre of the hexagon) were larger than the distances between the stigmatic lobes and the loci of the background color. In conclusion, due to the pollen, male flowers differed more from the leaves (background) than the female flowers and colour modelling suggests that honeybees can perceive this difference.

### Floral Scent

The average total amount of floral scent trapped in 12.5 min from eight male or eight female twigs used in the behavioural tests in 2007 (samples from cut twigs), was 665 (595–819) ng (Median, Min-Max) in males, compared to only 141 (85–144) ng in females (*Z*_7_ = 2.17; *p* = 0.030). An average of 8 (7–10) ng in males, and only 2 (1–2) ng in females (Z_7_ = 2.31; p = 0.021) was trapped per catkin per 12.5 min. Similar gender differences were found in the samples collected *in situ* in 2006: from a male catkin we trapped on average 12 (3–43) ng per 12.5 min and from a female catkin 2 (1–8) ng (Z_10_ = 2.56; p = 0.011).

We found in total 37 floral scent compounds, of which 36 occurred in samples of male flowering twigs and 34 in female flowering twigs (Table S1). Both sexes emitted nearly the same spectrum of compounds. Linalool, 1,4-dimethoxybenzene and methyl salicylate were the only compounds found in all 19 scent samples, followed by *(E)*-β-ocimene and phenylacetaldehyde which were detected in 17 samples. These most common compounds were also the most abundant compounds in the scent samples.

**Figure 4:**
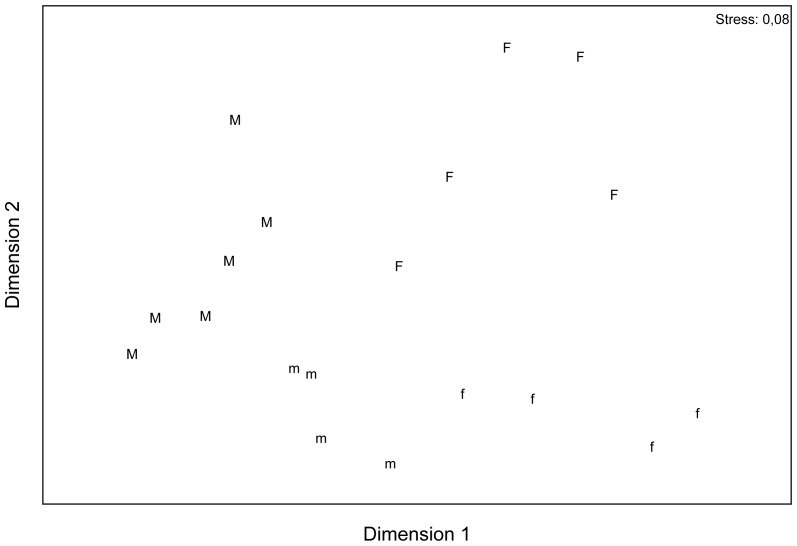
Nonmetric multidimensional scaling (NMDS) of floral scent composition (percentage amount) in *Salix caprea*. Female and male individuals of *Salix caprea* were sampled *in situ* in 2006 (F: females 2006, M: males 2006). Cut twigs were used in 2007 (f: females 2007, m: males 2007). Close samples (individuals) were similarly scented, whereas distant samples emitted a quite different scent pattern.

The similarity/dissimilarity of floral scent composition of female and male *S. caprea* (based on relative amounts of compounds) is demonstrated in Figure 4, using nonmetric multidimensional scaling (stress = 0.08). In general, we found significant differences between sexes (pseudo-F_1,15_ = 14.54, p<0.001) and this sex effect was the same in samples collected in 2007 from cut twigs and the samples collected 2006 *in situ* (sex×year: pseudo-F_1,15_ = 0.40, p = 0.90) (Figure 4).

According to SIMPER, four compounds (1,4-dimethoxybenzene, methyl salicylate, *(E)*-β-ocimene, and phenylacetaldehyde) explained more than 60% of the observed variability between male and female floral scent. While scent from female inflorescences was dominated by 1,4-dimethoxybenzene (48%) and methyl salicylate (15%), followed by *(E)*-β-ocimene (6%), the dominance of 1,4-dimethoxybenzene was much greater in males (71%). Methyl salicylate (7%) and *(E)*-β-ocimene (1%) were much lower in males than in females (Table S1). Basically, in both, samples collected *in situ* as well as from cut flowering twigs, 1,4-dimethoxybenzene was most responsible for the relative and absolute differences between the sexes; the weaker scent in females is mainly due to their lower emission of this aromatic compound.

### Bioassays

#### Visual and/or olfactory cues versus a negative control (Experiment 1)

**Figure 5:**
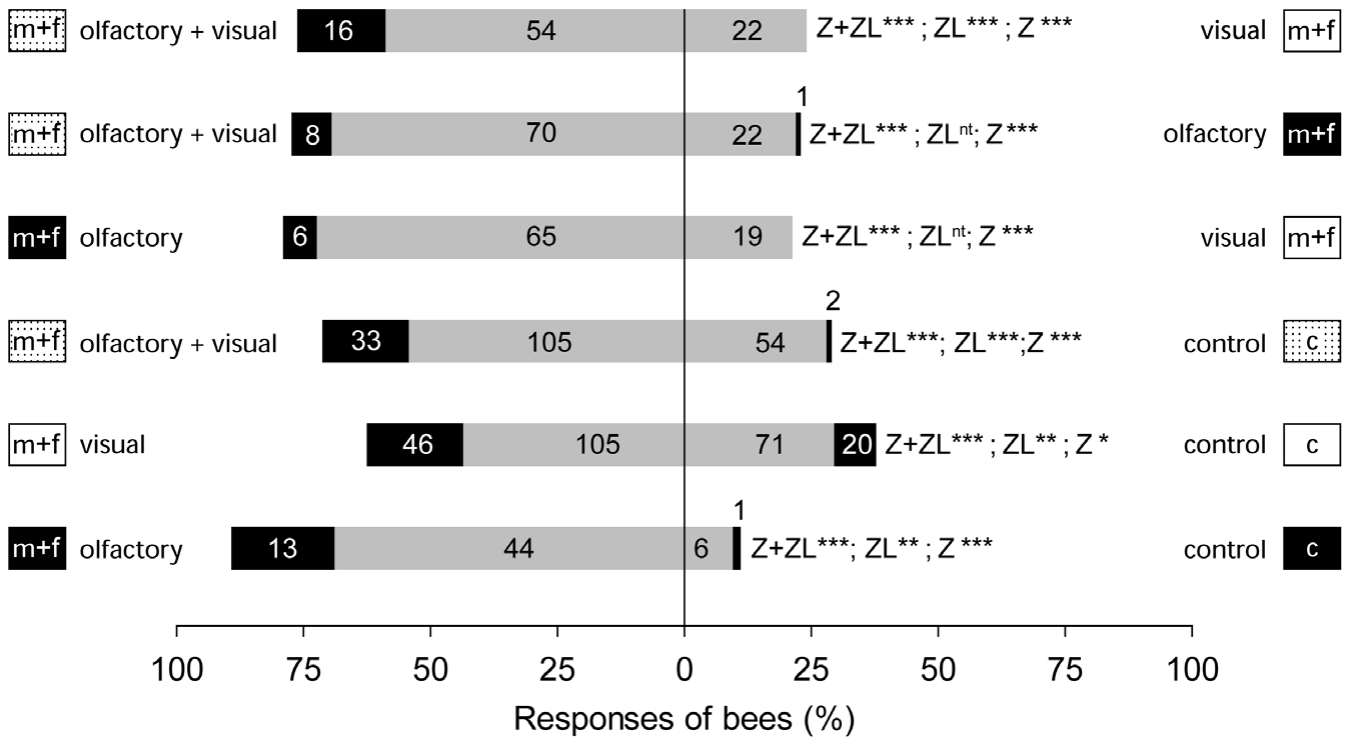
Responses of *Apis mellifera* to the olfactory and visual advertisement of *Salix caprea* flowering twigs. Olfactory and visual cues (alone or combined) of flowering sallow twigs were tested against an empty control cylinder (*Experiment 1*; lower three choice tests) or against each other (*Experiment 2*; upper three choice tests). Black = bees that landed after zigzagging (ZL); Grey = bees that zigzagged only (Z) without landing. The abbreviation “Z+ZL” refers to all bees that zigzagged with or without landing. The numbers in the bars indicate the absolute number of responding bees. Significant differences: *** *p*<0.001; ** *p*<0.01; * *p*<0.05, nt: a statistical test was not conducted due low number of bees. Symbols are as in Figure 2.

Olfactory and visual cues alone as well as the combination of both attracted more honey bees than the controls (Figure 5). In all three experiments more honey bees approached and landed on the cylinder loaded with female and male willow twigs than on the control.

#### Visual versus olfactory cues (Experiment 2)

Olfactory cues attracted significantly more honey bees than visual cues (Figure 5). The combination of both olfactory and visual cues was more attractive than either cue alone.

#### Gender comparison (Experiment 3)

**Figure 6:**
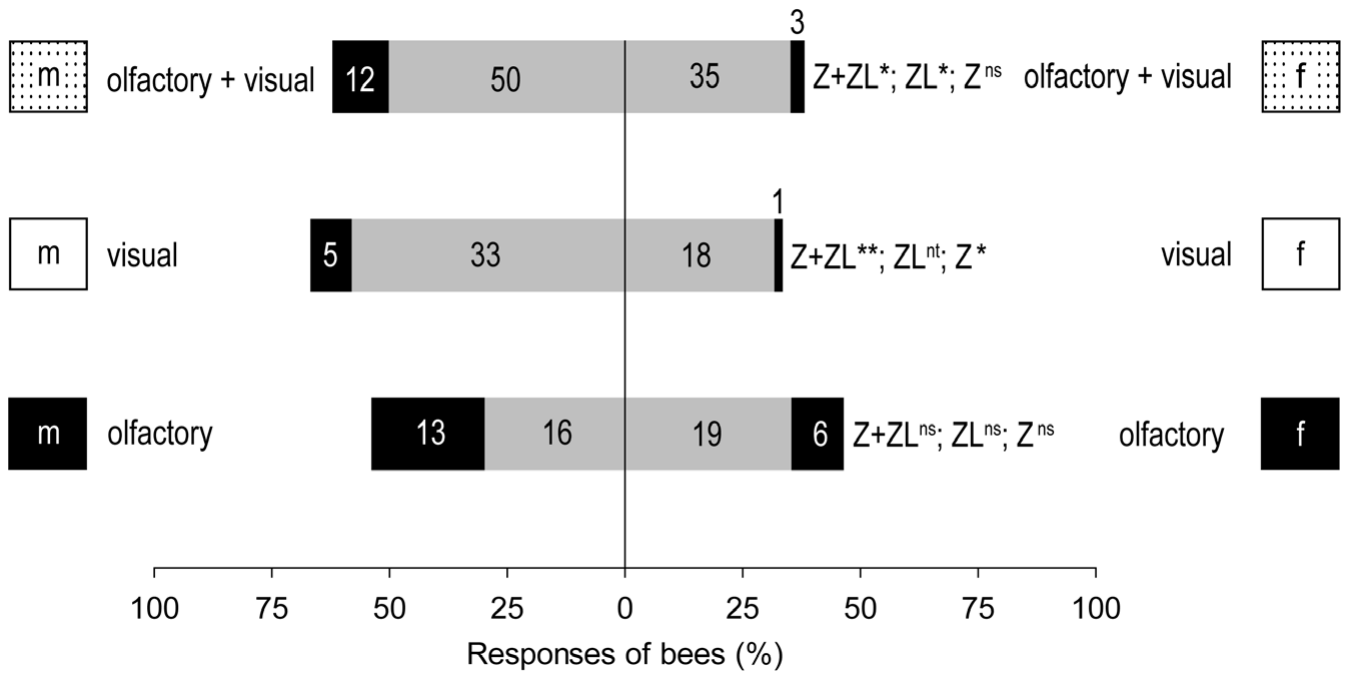
Attractiveness of female and male *Salix caprea* flowering twigs to *A. mellifera*. Olfactory and visual cues (alone or combined) of male flowering twigs of sallow were tested against the respective cues of females (*Experiment 3*). ns: p>0.05. Other symbols and abbreviations are as in Figures 2 and 5, respectively.

The odour of female and male flowers attracted nearly the same numbers of *A. mellifera* (Z+ZL) (Figure 6) and also the number of bees landing after zigzagging did not differ significantly. In response to visual cues however, significantly more honey bees approached and contacted the cylinder with male twigs than the cylinder with female twigs. Also, when combining both cues, male flowers were more attractive than female flowers, although the number of bees that approached without landing did not differ significantly.

## Discussion

### What is the relative importance of the visual and olfactory advertisement in attracting inexperienced honey bees to sallows?

Insects use both olfactory and visual cues to find flowers (e.g., [63]), and offering pollinators decoupled cues or a combination of both cues has shown that some insects respond more to visual [64]–[66], others more to olfactory [59], [64], [66] cues, and that in some insects a combination of both cues is needed to elicit specific behavioural responses [58], [60], [63], [67]. Our study shows that visual and olfactory cues are also important for naïve honey bees to find flowering sallow twigs. Bees innately respond to decoupled olfactory and visual cues of *S. caprea* (Figure 5), but olfactory cues were more attractive than visual cues alone, and the combination of both cues attracted more honey bees than either one alone (Figure 5). The stronger effect of scent compared to visual cues for attracting bees is consistent with earlier studies in other plant species and honey bees [68], [69] or other bee species [59], but examples in which the attractiveness of visual cues is higher compared to olfactory ones are also known in some bee species [58], [60]. Generally, flower scent is considered as an important cue for naïve bees (such as the sugar water-fed bees used in our study) searching for their first floral meal or for new food sources/patches [39], [40], [66], [70], [71].

In naïve honey bees, olfactory cues were described to be essential in eliciting landing responses [72], in our study however, the bees landed also when only visual cues alone were offered, and some even landed on negative controls (Figures 5 and 6). The different findings of both studies are likely due to methodological differences. While we used real flowering twigs, Giurfa et al. [72] used artificial paper flowers. Natural flowering twigs seem to be a better visual cue for honey bees than coloured paper, which only becomes attractive in combination with olfactory cues. In our study the test cylinders were located at the place where the sugar solution had been offered before the bioassays were conducted, and this may explain the landings on the negative controls (in smaller numbers compared to the treatments). Bees may have learned the location of the feeder and some may have landed in this region on any visual cue in expectation of food.

### Do female and male inflorescences have the same visual and olfactory advertisement?

In dioecious *Salix caprea*, to the human eye, the main difference between sexes can clearly be attributed to the presence of pollen. Male sallow inflorescences are yellow due to the anthers offering pollen whereas female catkins are inconspicuously greenish. Our colour modelling suggests that honey bees perceive similar differences between male and female flowers compared to the background of leaves. Although honey bees cannot discriminate the filaments and the ovaries from the background leaves, the stigmatic lobes and pollen have distinct colour loci from the colour locus of the leaves. The colour of pollen clearly differs from the colour of stigmatic lobes, and in the colour hexagon, the distances between the loci of the pollen colour and the uncoloured point (centre of the hexagon) were larger than the distances between the loci of the stigmatic lobes colour and the uncoloured point. Visual cues of flowering males are thus likely an honest signal (sensu [33]) for pollen availability.

Male *S. caprea* inflorescences emitted a higher total amount of scent (4–5 fold), a phenomenon described also from several other dioecious plants (reviewed by [15]). The higher amount of scent emitted by male inflorescences seems not to be just a consequence of bigger male catkins, as the dry weight of male catkins is only double the dry weight of female catkins (Glück, unpubl. res.). In contrast to this significant quantitative difference in scent, inflorescences of both sexes emitted nearly the same compounds. This confirms the results published by Füssel et al. [41] and Tollsten and Knudsen [73], who also found qualitative similarities in floral scent of both sexes of *S. caprea*. However, we detected differences in the proportions of scent components, which were not reported by Füssel et al. [41], although by Tollsten and Knudsen [73]. Male catkins emit more 1,4-dimethoxybenzene than females. We do not know whether male organs (filaments, anthers) are responsible for this finding, as we did not determine the contribution of the different inflorescence/floral organs or products (e.g., pollen) to the total scent by dynamic headspace. However, by thermally desorbing anthers with pollen in the injector of the GC (as described by [74]) we found in a preliminary analysis that they contain only a few compounds (10–14) with 1,4-dimethoxybenzene dominating the profile in most samples (Glück *et al.*, unpubl. res.). The pistil, which is known to contribute to the floral scent in other plants [75], seems to be of no importance in sallow as no compounds are emitted in considerable higher absolute amounts specifically in females.

### Do visual and olfactory cues of female and male sallows have the same attractiveness for inexperienced honey bees, and does nectar differ between female and male flowers?

Honey bees preferred male over female flowering twigs when relying on visual cues alone (Figure 6). This is not surprising considering the conspicuous colour of pollen, which clearly differs from female flower parts and green leaves. When male flowers are in full bloom, the pollen is available on the surface of the open anthers and the bright yellow colour is certainly readily perceived by the bees. Visual pollen cues have been known for a long time to elicit behavioural responses in honey bees [76]. Several plants, which do not offer pollen to the pollinators, possess flowers that mimic pollen colour cues to attract flower visiting insects, among them bees [77]–[79], pointing again to the importance of yellow colour cues (bee-green) for pollinators.

Despite differences in total scent emission (4–5 fold higher in males) and sex-specific differences in relative scent composition, naïve honey bees were equally attracted to the scent of both sexes, but it is unclear whether they are simply incapable to discriminate between the scents or whether that is a behavioural indifference. Bees, among them the honey bee, have receptors for most of the compounds responsible for sex differences in sallow, among them 1,4-dimethoxybenzene, which further is a potent attractant for the honey bee [39], [47]. We believe that honey bees are capable of detecting the differences in scent between male and female flowering twigs, being it due to the differences in relative scent composition or the total amount of scent emitted. Because of the higher amount of scent emitted by male catkins, bees may detect male flowering twigs from larger distances than female flowering twigs, and therefore be overall more attractive. Although observed differences in scent between the sexes did not result in a preference of naïve bees for either sex in our flight cage experiment (it did not test for a distance effect), honey bees visiting *S. caprea* for the pollen, may link the pollen reward with the scent of males, and thereby learn the differences in scent to avoid female plants when seeking specifically pollen. Honey bees are known to learn odours and also specific ratios of odours quickly [71], [80], [81]. This is also true for other pollinators and Ashman et al. [37] reported that in the gynodioecious *Fragaria virginiana* experienced pollinators (small bees) prefer hermaphrodite over female flowers primarily because of the scent of anthers, which emitted high amounts of 2-phenylethanol, a compound found only in small amounts in the female flowers.

In the field, visitation rates by the honey bee are more than double in male sallows compared to females (Susanne Kern, personal communication). Whichever attractive cue the bees use to distinguish male and female plants, the basic reason for differences in visitation rates in the field is most likely a result of the different rewards offered. Single honey bees visit flowers to collect nectar only, pollen only or both [82]. Male sallows therefore may be visited by bees collecting one or both products, whereas in females only nectar can be collected. The higher visitation rates to male plants therefore can easily be explained by the presence of pollen and the attraction of bees seeking pollen in particular. In addition, we found also sex-specific differences in nectar reward. The average amount (per flower in a sampled catkin) and the total concentration of nectar did not differ significantly between the sexes. This pattern is not only evident in open-pollinated flowers (as used in this study), but also in flowers, which were bagged to exclude visitors (Daniela Geis, personal communication). However, we found differences in the sugar composition with nectar of male flowers being sucrose-dominated, whereas the nectar of females is hexose-dominated. This pattern seems to be typical for *Salix* [83]. The three main nectar sugars occur in similar amounts in the nectar of females, a ratio that, according to Percival [29], is relatively rare in plants. Honey bees prefer nectars with more or less equal amounts of all three sugars over sucrose-dominated nectars [84]. For honey bees, nectar of female *S. caprea* may therefore be more attractive than nectar of males, however, apparently this cannot compensate for the lack of pollen, and does not affect the attraction of naïve bees as used in our bioassays. Overall, one sex does not invest more in sugar production than the other sex, but the females may nevertheless produce a more attractive nectar for honey bees, and bees may learn to distinguish male and female catkins and the specific rewards they offer: pollen/sucrose-rich nectar in males and hexose-rich nectar in females. It is unclear, whether the differences in relative sugar composition are a result of sex specific selection or of other ecological factors. Recently it was demonstrated that nectar properties are strongly influenced by nectar microbial communities [85], and a study on a dioecious species, *Silene latifolia*, revealed that the microbial community does not differ consistently between the sexes [86]. In *Salix* it is unknown, whether female and male flowers have different microbial communities, which may influence the nectar properties in the different sexes.

In sallow and in dioecious plants in general, it is essential that the pollinators visit males first and females second for pollination to occur. The higher attractiveness of males (as found in this and other studies with dioecious plants) directs the pollinators preferably to males, and ensures that they carry pollen from previous visits to males when visiting subsequently females. The pollen and/or nectar reward (depending on species) of male flowers could run short during the course of the day due to the high visitation rate, and pollinators may then switch to females, which still offer nectar, and act thereby as effective pollinators (see also [87]). Also, a difference in temporal pattern of nectar secretion between the sexes may induce such a shift, i.e. when males secrete nectar before females.

### Are the differences in the advertisement between male and female sallows a result of sex specific selection?

In willow species studied so far, pollen limitation seems to occur mainly in entirely insect-pollinated species [88], [89]. In *S. caprea* which has a mixed pollination system with insects and wind being pollinating agents, pollen limitation may be low and female fertility mainly resource limited. In consequence, male fertility in *S. caprea* may be more limited by access to mates than female fertility. As predicted by the sexual selection hypothesis males then should invest more in pollinator attraction than females. Our findings are generally consistent with this sexual selection theory; however, the visual conspicuousness of males may not be a result sex specific selection. Male flowering twigs are more attractive to naïve honey bees due to visual cues, and the yellow (bee green) colour of the pollen was most likely responsible for this effect. The higher attractiveness of males to honey bees is therefore simply achieved by the separation of the male and female function without investing additionally more in the visual display of male flowers. Pollen is yellow not only in sallow but in many flowering plants. Flavonoid pigments (which may be supplemented by carotenoids) are responsible for the yellow colour of pollen, and flavonoid-containing yellow pollen is not a specific adaptation for attracting pollinators [90]. In fact, pigments have their original function in protecting the pollen, e.g. from mutagenic UV-light [90]. However, especially in animal-pollinated plants, such as willows, which do not have a colourful perianth, pollen became an attractive cue for pollinators [90]. Overall, the higher attractiveness of males due to visual cues as found in our study seems to be simply the consequence of spatial separation of the androecia and gynoecia, and not of sex specific selection.

In contrast, the higher scent production (mainly 1,4-dimethoxybenzene) of male flowering twigs, might indeed be a result of sex specific selection. The increased emission of 1,4-dimethoxybenzene may have its primary (and original) function in attracting pollinators, and may have evolved under pollinator-mediated selection. Independent of whether the anthers/pollen or other floral structures are responsible for the higher amount of 1,4-dimethoxybenzene emitted in males, the differences between the sexes seem to be a result of sex specific selection in order to increase the attractiveness of males beyond the attractiveness gained from visual cues alone. An alternative hypothesis was discussed by Theis et al. [38]. They suggested that not only pollinator-mediated but also predator-mediated selection may play a role, leading to a lower amount of scent or a reduced visual advertisement in females compared to males in order to avoid attracting detrimental herbivores to females. In sallow, such detrimental insects may be *Egle* spp. (Anthomyiidae, Diptera). The adults of these flies lay eggs in flowering catkins, and the larvae feed on the growing seeds [48].

Further studies are now needed to explore the anatomical source of higher amounts of scent in males, how learning of advertisement cues and rewards affects foraging behaviour of experienced honey bees, and if pollinator- and/or predator-mediated selection shape sex-specific traits in *S. caprea*.

## Supporting Information

Table S1 **Occurrence and relative amount (%) of scent compounds emitted from female and male inflorescences of** ***Salix caprea*** **.**
(DOCX)
