# Supplementary material for: Floral Reward, Advertisement and Attractiveness to Honey Bees in Dioecious Salix caprea
Source: PLoS One. 2014 Mar 27;9(3):e93421. doi: 10.1371/journal.pone.0093421 (PMC3968154; doi:10.1371/journal.pone.0093421)
Supplement: Table S1 — Occurrence and relative amount (%) of scent compounds emitted from female and male inflorescences of Salix caprea . (DOCX) [file pone.0093421.s001.docx]

**Table S1:** Occurrence and relative amount (%) of scent compounds emitted from female and male inflorescences of *Salix caprea*.

|  |  | Male inflorescences | | |  | Female inflorescences | | |
| --- | --- | --- | --- | --- | --- | --- | --- | --- |
|  |  | Occurence^a^ | Median | Min-Max |  | Occurence^a^ | Median | Min-Max |
| ***Aromatics*** |  |  |  |  |  |  |  |  |
| Benzaldehyde |  | 5/4 | 0.4 | 0-0.9 |  | 5/4 | 1.7 | 0.2-13.0 |
| Benzyl alcohol |  | 6/1 | 0.4 | 0-1.0 |  | 5/0 | 0.8 | 0-3.4 |
| Phenylacetaldehyde |  | 5/4 | 0.5 | 0-6.0 |  | 4/4 | 3.4 | 0-9.6 |
| Salicylaldehyde |  | 2/1 | 0 | 0-0.4 |  | 0/1 | 0 | 0-0.2 |
| 2-Phenylethyl methylether |  | 3/3 | 0.1 | 0-1.2 |  | 0/4 | 0 | 0-0.8 |
| 2-Phenylethanol |  | 6/4 | 0.6 | 0.1-2.9 |  | 2/4 | 0.2 | 0-0.4 |
| 1,4-Dimethoxybenzene |  | 6/4 | 71.0 | 60.2-84.3 |  | 5/4 | 48.4 | 38.7-59.1 |
| Methyl salicylate |  | 6/4 | 7.0 | 1.9-8.4 |  | 5/4 | 15.2 | 7.2-19.8 |
| ***Isoprenoids*** |  |  |  |  |  |  |  |  |
| α-Phellandrene |  | 1/3 | 0 | 0-0.5 |  | 2/4 | 0.3 | 0-6.5 |
| α-Pinene |  | 0/4 | 0 | 0-0.2 |  | 0/2 | 0 | 0-0.5 |
| β-Pinene |  | 5/2 | 0.5 | 0-3.2 |  | 5/2 | 1.8 | 0-8.1 |
| β*-*Phellandrene |  | 0/3 | 0 | 0-0.1 |  | 0/1 | 0 | 0-0.3 |
| Limonene |  | 6/4 | 0.1 | tr^b^-0.4 |  | 4/4 | 0.3 | 0-1.9 |
| *(Z)*-β-Ocimene |  | 5/4 | 0.8 | 0-3.1 |  | 4/4 | 1.9 | 0-3.4 |
| *(E)*-β-Ocimene |  | 5/4 | 0.7 | 0-12.0 |  | 4/4 | 5.5 | 0-19.2 |
| Linalool |  | 6/4 | 3.9 | 0.6-13.1 |  | 5/4 | 1.9 | 0.3-13.6 |
| Lilac aldehyde A |  | 4/2 | 0.2 | 0-2.4 |  | 2/1 | 0 | 0-1.2 |
| Lilac aldehyde B+C |  | 4/4 | 0.3 | 0-2.3 |  | 3/4 | 0.3 | 0-0.6 |
| Lilac aldehyde D |  | 4/0 | 0 | 0-0.8 |  | 3/3 | tr | 0-0.6 |
| Lilac alcohol A |  | 2/0 | 0 | 0-0.2 |  | 0/0 | 0 | 0-0 |
| Lilac alcohol B+C |  | 2/4 | tr | 0-0.5 |  | 0/1 | 0 | 0-0.8 |
| Lilac alcohol D |  | 4/0 | 0 | 0-0.7 |  | 0/0 | 0 | 0-0 |
| α-Copaene |  | 0/3 | 0 | 0-0.1 |  | 0/0 | 0 | 0-0 |
| (*E*)-Caryophyllene |  | 0/2 | 0 | 0-0.1 |  | 0/1 | 0 | 0-0.1 |
| Geranylacetone |  | 6/4 | 0.2 | tr-1.7 |  | 5/3 | 0.2 | 0-3.6 |
| α-Cubebene |  | 0/4 | 0 | 0-0.1 |  | 0/2 | 0 | 0-0.1 |
| (*E,E*)-α-Farnesene |  | 3/4 | 0.1 | 0-0.9 |  | 4/4 | 0.6 | 0-7.5 |
| ***N-bearing compounds*** |  |  |  |  |  |  |  |  |
| Phenylacetonitrile |  | 5/4 | 1.0 | 0-11.8 |  | 4/3 | 0.5 | 0-6.8 |
| Indole |  | 4/4 | 0.6 | 0-1.5 |  | 5/4 | 0.7 | 0.4-3.2 |
| ***Fatty acid derivates*** |  |  |  |  |  |  |  |  |
| *(Z)*-3-Hexen-1-ol |  | 0/2 | 0 | 0-0 |  | 0/4 | 0.3 | 0-1.0 |
| *(Z)*-3-Hexenyl acetate |  | 4/4 | 0.2 | 0-0.9 |  | 4/4 | 1.2 | 0-2.2 |
| 4-Oxoisophorone |  | 5/2 | 0.5 | 0-11.8 |  | 3/4 | 0.2 | 0-0.6 |
| (*E*)-4,8-Dimethyl-1,3,7-nonatriene |  | 6/4 | 0.8 | 0.2-4.6 |  | 3/4 | 1.4 | 0-3.4 |
| ***Unknowns*** |  |  |  |  |  |  |  |  |
| m/z: 41, 45, 59, 73, 97 |  | 6/3 | 1.0 | 0-6.8 |  | 5/4 | 2.1 | 0.9-6.2 |
| m/z: 39, 77, 91, 119, 134 |  | 0/4 | 0 | 0-0.2 |  | 0/4 | 0 | 0-0.5 |
| m/z: 40, 55, 69, 119, 154 |  | 0/4 | 0 | 0-tr |  | 0/4 | 0 | 0-tr |
| m/z: 40, 55, 95, 123, 138 |  | 0/2 | 0 | 0-0.1 |  | 0/2 | 0 | 0-0.4 |

^a^: Occurrence: number of plants sampled in 2006/2007, where a specific compound was found. Total sample sizes: Male inflorescences 2006/2007, n = 6 / n = 4; Female inflorescences 2006/2007, n = 5 / n= 4.

^b^: trace: amount was less than 0.05.
